# Impact of CRISPR-Cas9-Based Genome Engineering in Farm Animals

**DOI:** 10.3390/vetsci8070122

**Published:** 2021-06-30

**Authors:** Parul Singh, Syed Azmal Ali

**Affiliations:** Proteomics and Cell Biology Lab, Animal Biotechnology Center, ICAR-National Dairy Research Institute, Karnal 132001, India; parulbiotech92@gmail.com

**Keywords:** genome editing, ZFNs, TALANs, CRISPR-Cas9, guide RNA, livestock, precision, specificity

## Abstract

Humans are sorely over-dependent on livestock for their daily basic need of food in the form of meat, milk, and eggs. Therefore, genetic engineering and transgenesis provide the opportunity for more significant gains and production in a short span of time. One of the best strategies is the genetic alteration of livestock to enhance the efficiency of food production (e.g., meat and milk), animal health, and welfare (animal population and disease). Moreover, genome engineering in the bovine is majorly focused on subjects such as disease resistance (e.g., tuberculosis), eradicate allergens (e.g., beta-lactoglobulin knock-out), products generation (e.g., meat from male and milk from female), male or female birth specifically (animal sexing), the introduction of valuable traits (e.g., stress tolerance and disease resistance) and their wellbeing (e.g., hornlessness). This review addressed the impressive genome engineering method CRISPR, its fundamental principle for generating highly efficient target-specific guide RNA, and the accompanying web-based tools. However, we have covered the remarkable roadmap of the CRISPR method from its conception to its use in cattle. Additionally, we have updated the comprehensive information on CRISPR-based gene editing in cattle.

## 1. Introduction

**Figure 1:**
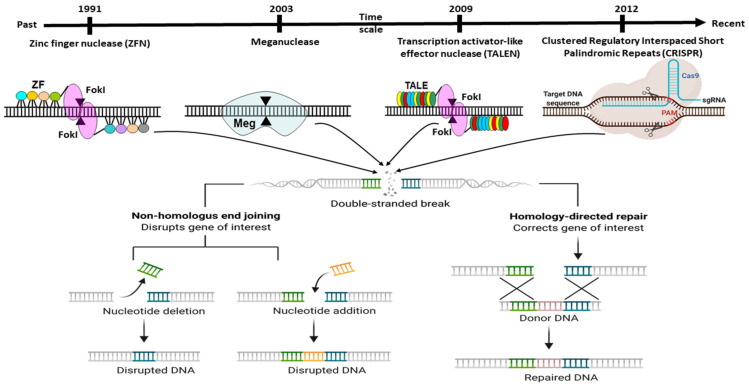
Diagrammatic illustration of various genome editing methodologies which is possible since the evolution of multiple precise molecular techniques. They work as exact molecular scissor, majorly, ZFNs (Zinc finger nucleases), TALENs (Translation activator-like effector nucleases), MegNs (Meganucleases), and CRISPR-Cas9 (Clustered regularly interspaced short palindromic repeats-CRISPR-associated system). Here, ZF: Zinc Finger, and PAM; protospacer adjacent motif.

Genome editing is the captivating genetic engineering approach with enormous potential in the biomedical application of gene function manipulation. It ensures the ability to treat or anticipate various genetic disorders through deletion, addition, or base change at a specific location of the desired organismal genome’s gene of interest (GOI). The ideal genome-editing approach needs to effectively alter a genomic sequence, showing higher DNA sequence specificity with less or no off-target effects. The strategy of genome engineering has to possible change genomic sequence, also should have higher DNA sequence specificity with fewer or no off-target effects. The idea of genome engineering begins with the enhancement of several specific molecular tools. They work as precise molecular scissor, known as Zinc Finger Nucleases (ZFN) [1], Translation Activator-Like Effector Nucleases (TALENs) [2], MegNs (Meganucleases), and CRISPR-Cas9 (Clustered Regularly Interspaced Short Palindromic Repeats (CRISPR)/CRISPR-associated nuclease (Cas) 9) [3,4,5] (Figure 1).

CRISPR-Cas9, a bacterial antiviral framework, is the recently developed modern era of technology with gigantic potential capabilities. Shockingly, the thought of the strategy is motivated and adaptive from the single-celled microscopic organisms (bacteria) and archaea [6], where this life forms a utilisation endogenous CRISPR system as the versatile immune strategy. In essence, this is a defence mechanism against viruses or other pathogens’ genetic sequences [7,8,9,10,11,12]. Moreover, these microbes are specialised in building up heritable memory of past assaulted phage or other pathogens through this strategy to cut up and devastate invader’s DNA in peace and long-term prospects [13].

## 2. Adaptation of Adoptive Mechanism as CRISPR Editing System

CRISPR–Cas mediated immune response in microscopic organisms is noteworthy and comprises three mechanistic steps: spacer acquisition/adaptation, crRNA (CRISPR RNA) biogenesis/expression, and target interference. The molecular mechanism is specified all the way through each level; in the first stage, microbes capture a part of the hereditary/genetic material of viruses and integrate it as the primary spacer into the CRISPR cluster. In this way, it permits bacteria to remain immune against viruses or closely related ones in the future through making a genetic memory. During crRNA biogenesis, rehashed viruses’ assault triggers the entire CRISPR cluster expression to specific pre-crRNA (pre-CRISPR RNA), which assists into mature crRNA by ribonuclease RNase III and taken after binding with trans-activating crRNA (tracrRNA) through a direct repeat. Each crRNA contains a distinctive sequence for target interference; all crRNA and tracrRNA make a complex with Cas nuclease protein to form a ribonucleoprotein effector complex. The crRNA acts as a guide to superintend this effector complex to impair the viruses [8,14,15]. Few studies have shown ‘how the prokaryotic CRISPR–Cas system can be utilised as a perfectional and exact molecular scissor after a couple of manipulations in crRNA’, since single guide RNA (g-RNA) replaces the necessity of both the crRNA and tracrRNA. Therefore, effective gene editing through CRISPR employs two critical components: a g-RNA and the Cas9 protein [16,17].

For a long time, transgenesis in mammalian cells and especially embryos contains hurdles, mainly for large animals such as livestock. Since the discovery of the engineered nucleases adopted allows us, by adding a site-specific double-stranded break (DSB), to make precise the genetic manipulation of specific genes or sequences by means of HR (Homologous Recombination) and NHEJ (Non-Homologous End Joining) repair pathways [18]. However, site-specific Cas9 generated DSB effectively stimulated the HR pathway approximately 10,000-fold in the lower organism [19,20]. In contrast, the competitive NHEJ route for DSB repair, is routinely favoured and leads, as much as possible, to minor insertions or deletions (indels) in mammals [21,22]. For the development of biomedical models, therapeutic trials, and joint breeding, site-specific genome manipulation is a critical method. Although preliminary research on the use of engineered nucleases for precise genetic engineering of food animal species focused on ZFN [23,24,25,26,27], meganucleases [28], and TALENs [29,30,31,32]. Later, CRISPR/Cas9 gradually emerged as the tool of choice due to its easy architecture and implementation [33,34].

**Figure 2:**
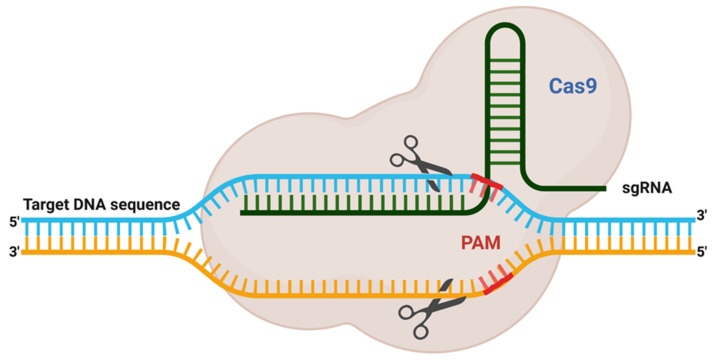
This picture is depicting the adopted functional CRISPR complex containing single guide RNA and Cas9 protein. It is the reconfiguration of natural dual RNA (tracrRNA and crRNA) system to a single-guide RNA (sgRNA) of 19–24 bp, which is good enough to program Cas9 to introduce DSB in target DNAs in vivo. PAM; protospacer adjacent motif.

To begin, gene editing technology was developed in the late twentieth century and is still evolving. Despite this, the whole subject has garnered considerable attention since its discovery. The first gene editing technique that established a foundation in the area of recombinant DNA technology was ZFN, launched in 1991 and widely utilized for many decades [35]. Additionally, another gene editing technique known as TALENs was developed in 2009 in response to the discovery of the genome-targeting capacity of TAL effectors (TALE) [36]. Later that year, another interesting genome modification technique, termed CRISPR, was discovered (Figure 1). It synthesizes a combination of short directed RNAs (guide RNA) and Cas-9 nuclease and is forced to build a tailored endonuclease for each target, a need that TALEN and ZFN cannot meet (Figure 2). Since this discovery, the entrance barrier to genome editing has been substantially reduced, allowing for more user participation and creativity [36]. The CRISPR/Cas9 protein complex (tracrRNA) requires two RNA transcripts: the crRNA and the trans-acting CRISPR RNA [37,38]. When this dual RNA restriction is reconfigured as a single-guide RNA (sgRNA) of 19–24 bp, Cas9 is functional and effective in generating DSB into the target gene’s DNA sequence [37].

Starting reports with the CRISPR/Cas9 system promised [5,39] and quickly amended for genome manipulation in numerous cells of diverse species, even for large animals [40,41]. Genome editing in goats and pigs is now generated efficiently using SCNT (somatic cell nuclear transfer) through CRISPR/Cas9-mediated edited cells serving as donors [42,43,44]. In genome-edited mice, rodents, monkeys, dogs, goats, pigs, and rabbits, a different direct method, consisting of direct injection in the cytoplasm of one-cell embryos, was discovered [34,45,46,47,48,49,50,51,52,53,54,55]. Editing efficiency in pigs ranged from 63 percent, while in goats, it ranged from 15 percent to 21 percent [46,50]. Injecting CRISPR/Cas9-associated RNA into zygotes will cause mosaicism in the long run [50,51,52,53,56]. Knocking out genes with CRISPR technology is a one-step procedure that involves microinjection at the zygotic level.

For this intention, CRISPR is directed to the initial coding region of the targeted gene of interest, creating DSB. This DSB will repair either mechanism (HR or NHEJ), which establishes an indel (insertion or deletion) mutation at the targeted region of the gene. Created indel serves as a stable mutation, since it is made through CRISPR and can produce knock-out alleles [57]. Despite the potential that CRISPR innovation might have in cattle, few reports are accessible so far.

## 3. Bioinformatics Tool Used to Design sgRNA for Gene Editing

**Figure 3:**
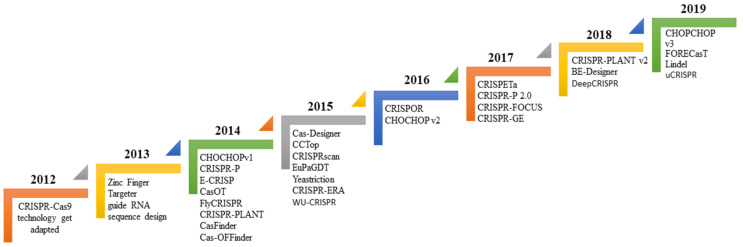
Time scale-based evolutionary representation of various Web-based sgRNA design tools from the past year to present.

As per previous studies, CRISPR/Cas9 protein recognises PAM sequence, sgRNA act to help to identify target loci followed by activation of endonuclease activity to cleave at a specific site. Cas9 enzyme cleavage activity varies significantly among different locations and cell types, owing to several factors that can affect the linking and cleavage potential of the sgRNA–Cas9 system. Therefore, various investigations have revealed that all included guide RNA characteristic (like composition, position and GC content), physical attributes (like melting temperature, and secondary structure formation) and chromatin remodelling for differential gene expression, together affecting the sgRNA efficiency. Various characterising tools were created to design highly efficient guide RNAs (Figure 3).

## 4. Guide RNA Sequence Features

Target sequence nucleotide constitution is one of the concerning factors of sgRNA efficiency and specificity for the genome editing activities by Cas9 [58]. The broad-scale screening of CRISPR-based editing in mammals demonstrated that cytosine is more favourable at the cleavage position (-3 position proximal to PAM [59]. Similarly, guanine is most advantageous at site 1 and 2 ahead of the PAM sequence, whereas GC content of the downstream sequence of the PAM region, especially 4–13 bases, come up with sgRNA efficiency. Contrarily, thymine is not likely preferred at +/−4 nucleotides which neighbours the PAM [60].

However, sequence upstream to PAMs sequence may not influence sgRNA efficiency. The downstream line, on the other hand, is expected to have a major impact on efficiency [61]. Based on this valuable information, various efficiency models have been generated. The energetics related to the emergence of the guide RNA, DNA, and Cas protein complex are customary and might elucidate to eliminate biases between distinctive models, because a few energetics approaches may better outline the Cas nuclease editing effectiveness [61,62,63]. Furthermore, other factors, such as genetic and epigenetic properties, including gene position, chromatin accessibility, and expression, are also essential constraints that influence Cas nuclease activity and sgRNA binding [61]. However, various studies have investigated that nucleosomes negatively affect Cas9 target cleavage activity; on the other hand, DNase I hypersensitivity and epigenome markers affect guide RNA efficacy [64,65]. Keeping all property mentioned above and their effects on efficiency, numerous computational tools for evaluating guide RNA efficiency and prediction of its specificity have been created so far (Table 1).

Hence, mostly existing guide RNA design tools have centred essentially on the choice of guide RNAs with high specificity for targeting the genome (on-target effect). However, before deciding on the best guide RNA, the off-target effects of the CRISPR-Cas9 system should be considered. Moreover, examining the target site is often a little easier by scanning the PAM sequence that “NGG” specific for the CRISPR/Cas9 from S. pyogenes. Challenge comes into consideration when we design guide RNA considering cleaved efficiency and specificity (no off-target effect). Therefore, a major hurdle for CRISPR application is its off-target effects. CRISPR nucleases can cleave unintended at the non-targeted genomic location and cause unforeseen nucleotide alteration or mutation due to guide RNAs recognising genomic DNA sequences with some mismatches or DNA/RNA bulges, which is called an off-target effect [22,66]. This drawback can be overcome through effective cutting by forecast CRISPR cleavage specificity and by designing ideal guide RNAs [61]. Therefore, ideal guide RNAs should contain high efficacy with excellent specificity [62]. Moreover, to assist researchers in choosing the most excellent guide RNAs for input DNA sequences, it is mandated to recognise guide RNAs with potential off-targets and precisely predict their relative cleavage rates. Various computational tools have been developed to facilitate sgRNA designing purposes [62].

**Figure 4:**
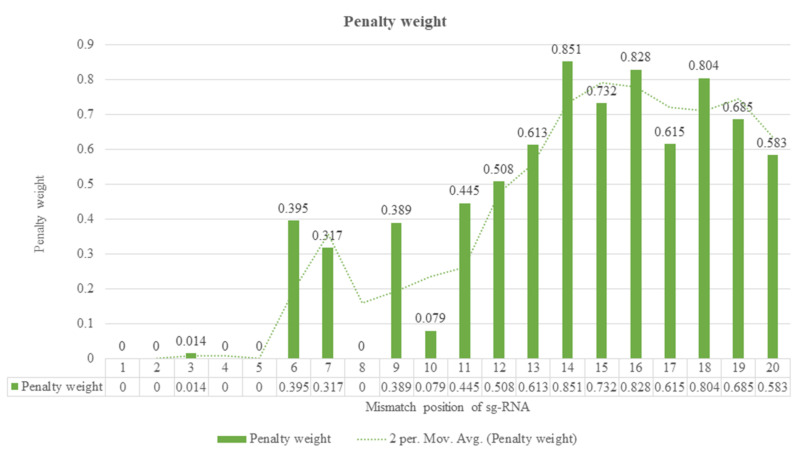
Graphical representation of the effect of the number of mismatches and their position of sgRNA on hypothetic cleavage rate. This graph shows penalty weights (0–1) to capture the position-dependent mismatch that impacts cleavage activity, where 0 implies no mismatch impact, and 1 shows the most significant effect on cleavage.

The Zhang laboratory has been investigating the effect of the number of mismatches and their position on guide RNA with hypothetic cleavage rate, using >700 guide RNA variants by targeting 15 genomic regions in a human cell line [22]. As a result, several guide RNA variants were created to account for all potential outcomes for single-nucleotide mismatches. A subset of various mismatches was transfected into cells, and the cleavage frequencies were compared to the related guide RNAs by using high throughput sequencing to sequence a genomic region multiple times for each target site. It comes to light that both the number and position of mismatches affect the activity of sgRNA. For example, a mismatch at positions ‘1–5’ (close to PAM) has almost no effect on cleavage activity, while mismatches at positions ‘13–20’ significantly affect activity (Figure 4) [22].

The above shows the penalty weights (0–1) to capture the position-dependent mismatch impact on cutting operation, with 0 indicating no mismatch impact and 1 showing the most critical effect on cutting activity. A foremost step for CRISPR-mediated gene editing is designing target-specific guide RNA. Single guide RNA plays a crucial function in recruiting Cas9 to a target site on the genome. There are numerous web-based tools available for designing of target-specific sgRNA; each of them has their advantage and disadvantage. The information we have provided in this review will help you to choose the best tool based on your requirement [61,67,68,69,70,71,72,73].

These tools promote the creation of guide RNAs to minimise off-target effects (high specificity) while optimising on-target efficacy (high sensitivity). For guide RNA on-target identification and efficacy prediction, several guide RNA designing tools have been developed. These strategies are classified into three types, (1) alignment-based (e.g., CasFinder), (2) hypothesis-driven (e.g., E-CRISP, CHOPCHOP, CRISPR, GuideScan), and (3) learning-based (e.g., sgRNA Originator, SSC, sgRNA Scorer, CRISPRscan) [71]. Generally, web-based guide RNA design tools needed users to input targeted DNA sequence, target gene name, or target genomic location with the species name. An algorithm specific to all tools output provides a list of candidate guide RNA sequences comparing predicted off-target sites for each input. Many of them are targeted to provide a guide RNA sequence that minimises the likelihood of off-target effects, but their strategy can be varied, specifically in CHOP-CHOP, which uses empirical data from multiple recent articles to compute efficiency scores [67,70].

Similarly, other tools like CasFinder and E-CRISP inculcate particular user-defined penalty conditions on position and a number of mismatches correspond to guide sequence to rank the potential for off-target effects [74]. However, some tools are designed for a specific application. CRISPR-ERA is the only tool that design guide RNA for gene repression or activation [75,76]. Meanwhile, FlyCRISPR concentrates on applications in beetle, fly, and worm species, such as the prevalent model organisms Caenorhabditis elegans and Drosophila melanogaster [77]. Furthermore, Benchling is a singular one that can design target-specific guide RNA that are compatible for both alternative nucleases, including *Staphylococcus aureus* Cas9 and Cpf1 [76,78,79,80,81,82] (Figure 3).

## 5. A Different Mechanism for the Transport CRISPR System

**Figure 5:**
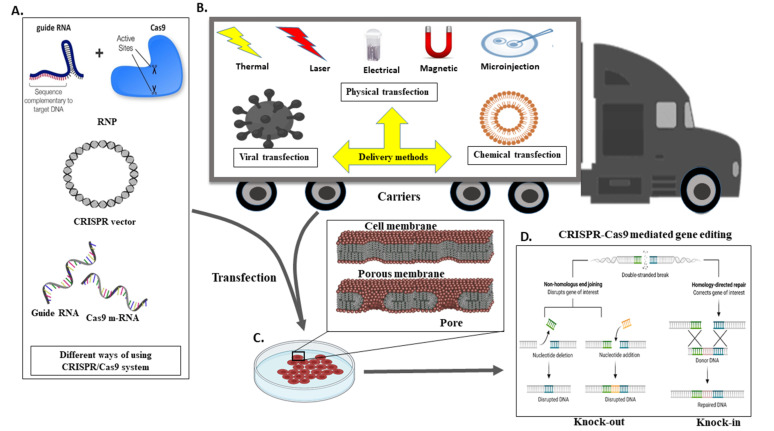
Different Methods of Delivering CRISPR/Cas9 into Cells. Schematic demonstration of in vivo CRISPR/Cas delivery modes and vehicles in numerous biological frameworks. Frameworks utilised to deliver CRISPR/Cas components can be separated into two major categories, CRISPR/Cas delivery mode and delivery carrier. (**A**) Three CRISPR/Cas delivery models, including protein (Cas protein with guide RNA as a ribonucleoprotein complex, RNP), DNA (plasmid encoding both the Cas protein and the gRNA), and RNA (mRNA for Cas protein translation and a separate gRNA), (**B**) Can be delivered into mammalians, aquacultures or plants by means of bacterial or viral vectors, chemical and physically directed delivery method, (**C**) To facilitate the delivery of the CRISPR system in the cell, transfection is accomplished by creating a membrane pore, and (**D**) Through the CRISPR framework, indel creation (knock out) or knock-in of a gene of interest in a targeted cell is possible.

The delivery of Cas9 into cells is an imperative thought in gene editing. Adopted CRISPR–Cas can be utilised in different ways or formats, for instance, m-RNA (direct transfection of sgRNA and Cas9 RNA), DNA (vector-based strategy), and in the form of RNP (ribonuclease protein complex). For detailed information around the CRISPR-Cas transport systems, it would be ideal to follow the recent review article by Lino et al. [68,83] (Figure 5A).

The delivery method for CRISPR is very much similar to the standard transfection method for nucleic acid. CRISPR/Cas9 system delivery inside the cell usually conducts through either viral or chemical processes. Generally, physical processes are taken on electrical or mechanical forces to form transient pores in the membrane of cells, facilitating the update of CRISPR molecules. Moreover, recently, due to nanotechnology and microtechnologies, the physical method for transfection is in higher demand. For instance, nanostructure-mediated electroporation permits miniaturisation or shortened the physical transfection method to enhance transfection efficiency and precision [68]. It has the advantage that it homogeneously treats cells with more minor or no viability damage to cells than bulk electroporation. Usually, CRISPR/Cas9 protein complexes have to be delivered in the cytoplasm of the transfected cells. To achieve efficient gene editing, CRISPR/Cas9 protein complexes must cross both the cell membrane and the nuclear membrane. As a result, the nuclear localisation sequence (NLS) directs the CRISPR/Cas9 system to the nucleus-encoded by the plasmid vector or the Cas9 protein. In the absence of an NLS sequence or signal, the CRISPR/Cas9 complex only enters the nucleus at the time of cell division when the membrane is disrupted [68].

Different transfection methods include viral transduction, in which a viral vector is used to transfer the CRISPR/Cas9 construct into the host cell. Viruses commonly used for viral transductions are lentiviruses, adeno-associated viruses (AAV), adenoviruses, and retroviruses. On the other hand, chemical transfection uses chemical mediators such as lipid vesicles and polymer-based chemicals to deliver the CRISPR/Cas9 construct to the target cell. Furthermore, the physical method is independent of the vector. Unlike viral vectors, it has no size restrictions. Furthermore, unlike the chemical process, it does not have a rate-limiting phase independent of cell endocytosis. Whereas physical transfection, using energy from thermal, electrical, and mechanical forces. As these forces are applied to cells, they weaken the cell membrane and build a pore, allowing the CRISPR/Cas9 construct to enter the cell or assist in the construct’s active delivery. Consider electroporation, which shocks the cell with an electric field, causing membrane perforation, while drifting forces to charged constructs or cargo, such as plasmid DNA, cause membrane perforation (Figure 5B).

## 6. Genome Editing in Ruminants Such as Cattle and Buffalos

It has been anticipated that in the population of 7.6 billion humans globally, every ninth individual (821 million people) does not have sufficient food to cover an active life [81]. Despite the lack of food, the human population is expected to rise to 8.5 billion in 2030, 9.7 billion in 2050, and 11.2 billion in 2100 [82]. As a result, the United Nations’ Food and Agriculture Organization (FAO) predicts that total agricultural yield (crop yield and animal-based products) should rise to 60% to fulfil global demand. More specifically, this percentage is further contributed to by animal protein, such as meat production by 76%, and milk productivity will need to increase by 63%. In order to achieve this ultimatum goal, a precise and practical approach should be used [84]. Meanwhile, genomics targets for genome engineering are possible by screening the differential expression using high throughput proteomics or genomics techniques [85,86,87,88]. In this regard, the generation of collective knowledge across the globe allows one to share and build the more efficient farm animals breeds [89].

Genome-editing and transgenic innovations offer the chance for more significant gains over a shorter time. Until now, genome editing investigation in cattle has centred fundamentally on enhancing the efficiency of food productivity (e.g., meat and milk), animal health, and welfare (animal population, surveyed or hornlessness and disease), generate all-male offspring, eradication of allergens from products (e.g., beta-lactoglobulin knock-out). On the other hand, genome editing might be utilised to precisely knock-in valuable alleles (such as heat tolerance, illness resistance), as well as haplotypes into our native locally well-adapted cattle breeds genome, subsequently to improve their productivity [90]. We recently used the buffalo mammary epithelial cells to understand lactogenic signalling [91,92].

Early research was majorly focused on animal growth. Skeletal muscle gives meat for human utilisation or consumption, consisting of muscle fibres, intramuscular adipose tissues, and connective tissues [93]. The importance of growth hormone (GH) and insulin-like growth factor I (IGF-I) in regulating body size in developing animals has long been recognised. GH and IGF-I play an essential role in muscle growth, both before and after birth [94]. The GH–IGF axis (growth hormone-insulin-like growth factor axis), regulated by the pituitary gland and liver, is responsible for muscle growth and body mass [76]. GH, on the other hand, induces the development of IGF-I in almost all tissues. The liver is the only organ that can primarily produce serum IGF-I. The pituitary gland produces GH, which stimulates the development of IGF-I in other tissues (liver and muscle). Even though some cIGF-I is released from other tissues, such as muscle, the liver is the most common source of circulating IGF-I (cIGF-I). cIGF-I is a component of the negative feedback loop that controls GH secretion [94]. IGF-1 derived from both muscle and liver plays a crucial role in myogenesis [95]. At the same time, a mutation in the IGF-2 gene’s regulatory function has been linked to increased muscle growth in pigs [96]. In recent years, effective microinjection of the GH and IGF-1 genes into pig zygotes has been reported. Later, two lines of GH-expressing transgene pigs gained 11.1 and 13.7 percent more mass than control pigs [97,98,99]. When transgenic technology is combined with recent genome editing technology, it creates a new age or property for animal protein that could affect animal welfare, while meeting human diet demands. The cloned pig, for example, that expresses the fat-1 gene from the nematode C. elegans, has a lower ratio of n–6 to n–3 fatty acids. A higher ratio of n–6 to n–3 fatty acids has been linked to poor bone health in humans. A lower ratio is related to healthier bone properties; thus, reducing both fatty acids can have nutritional health benefits in a diet [100].

Furthermore, related modifications have been observed in pigs containing the C. elegans n–3 fatty acid desaturase gene (encoded by the fat-1 gene) [101,102,103]. Similar findings were obtained when CRISPR/Cas9 was used to insert the fat-1 gene into the pig in the rosa 26 locus [104]. This is in proximity with gene alteration (genetic manipulation), which depends on the internalisation of the artificial gene (transgenes) to improve characteristic traits in animals. The genome/gene editing method allows us to make precise and error-free modifications to a livestock animal’s genome, to increase productivity, production, and infection resistance. In the genome editing region, targeted gene editing of the myostatin gene is a popular goal for increasing growth and muscle production. They were first noticed in heavily muscled sheep and cattle like Piedmontese and Belgian Blue cattle and the Texel sheep breed. Additionally, it was discovered that decreased expression of the myostatin gene (also known as GDF8, or growth differentiation factor 8) results in increased muscle growth. Single-nucleotide polymorphisms in the myostatin gene trigger a fundamental genetic change. The Piedmontese and Belgian Blue have a single-nucleotide polymorphism in the myostatin gene and an 11-bp deletion in the myostatin gene [104,105,106].

Currently, a large number of genome-edited animals have been developed, including an MSTN gene-edited pig, an anti-PRRS gene-edited pig, and tuberculosis-resistant transgenic dairy animals (Cattle) [107,108,109]. This genetically modified/edited livestock has shown significant improvements in meat yield, disease tolerance, and other desirable traits [106]. Genome modification technique (especially ZNFs and TALENs) in cattle has been continuously growing over time. For instance, targeted gene editing through ZFN technology has been performed in cattle to generate gene-edited cattle for ZFN-mediated β-lactoglobulin gene mutation [110]. Furthermore, it has been demonstrated that introducing the human lysozyme gene into the cattle β-casein locus in mammary cells results in transgenic cattle that can secrete human lysozyme in their milk to suppress microbes such as *Staphylococcus aureus* [27,110].

TALEN technology, on the other hand, was used to insert the SP110 gene into the cattle genome, resulting in tuberculosis-resistant transgenic cattle [111]. Furthermore, the β-lactoglobulin (LGB) gene in cattle embryos has been altered with remarkable effectiveness utilising the ZFNs and TALENs approaches [112]. MSTN gene knock-out cattle have been generated using TALEN [32]. TALENs were also used to eliminate the POLLED allele in Holstein cattle and produce hornless dairy cattle [113].

## 7. Methods for Generating Large Animals from CRISPR Edited Genomes

In the past 35 years ago, genetically engineered livestock had been generated by microinjection of interest DNA into the pronucleus of zygotes [114]. However, numerous genetically modified animals were developed in various species, such as livestock like goats, pigs, cattle, sheep, and rabbits, by injecting gene of choice into the pronucleus of a zygote [115,116]. Both direct editings of zygotes and somatic cell nuclear transfer (SCNT) from fibroblasts showed significant success in the field of editing. In either case, based on the success rate of the older transgenesis editing process, modification in spermatogonial stem cells may be a third viable option.

The SCNT approach was initially developed in Dolly the sheep in 1996 by Wilmut et al. However, a similar approach was later demonstrated in livestock species including cattle, goats, pigs, and equine, which provides the first cell-mediated interface for livestock genetic engineering; this is intriguing [117,118,119,120]. In SCNT, oocytes are typically harvested from slaughterhouse ovaries, produced or matured in vitro, and enucleated and melded with the nucleus from the gene-edited cell. Increased time in culture during the gene editing process does not appear to reduce SCNT’s efficacy. Furthermore, cloning was affected by poor performance and well-being issues in first-generation clones about 20 years ago; but, with advancements in science in today’s world [121].

Injection of CRISPR/Cas9 constructs or some other probabilistic process directly into zygotes has been shown to produce gene editing progeny in direct zygote editing. In terms of time and avoiding unwanted future cloning defects, direct editing of the zygote has several advantages over the traditional SCNT process. CRISPR/Cas9 construct with the ability to disrupt gene and introduce site-specific mutation precisely has been directly injected into zygote for zygote manipulation (cytoplasmic injection, or pronuclear or electroporation) [122]. Moreover, the non-stationary nature of the NHEJ course is quite random, which is suitable for large litter animals where helpful edits can be selected [121]. However, genetic mosaicism is a significant challenge using the zygote manipulation approach, especially when the resultant offspring shows both germline and somatic traits. As a result, one of the suggested ways to resolve genetic mosaicism is the direct injection of the CRISPR/Cas9 cascade during metaphase II (MII) oocyte or a very early zygote level. Initial zygote phase electroporation with that of the Cas9-RNP combination, for example, completely removed the mosaic mutation [123,124]. In sheep and cattle, injection of CRISPR/Cas9 into zygote injection is more efficient to reduce mosaicism compare to MII oocytes injection [57,125].

In 2013, a viral vector containing GFP was implanted into spermatogonial stem cells, then transplanted into boars [121]. Transgene expression was found to persist in sperm for up to five years, allowing for the effective and stable development of transgenic embryos via IVF. When it comes to the fact that there are still technological barriers to using current gene editing techniques rather than viral strategies (specifically, the ability to produce spermatogonial stem cells in culture), spermatogonial stem cell transfer may be a viable alternative approach for using cutting-edge gene editing methods to create large animals [121].

## 8. CRISPR Cas9 Mediated Genome Editing in Cattle and Buffalo

Genome engineering in the bovine is majorly focused on subjects such as disease resistance (e.g., tuberculosis), eradicating allergens (e.g., beta-lactoglobulin knock-out), product generation (e.g., meat from male and milk from female), male or female birth specifically (animal sexing), the introduction of desirable phenotypes (e.g., stress tolerance, disease resistance) and their long-term survival (e.g., polled or hornlessness) [99]. Till 2015, a dependable tool for genome altering was inefficiently and not routinely utilised. Since the CRISPR/Cas9 set foot in the gene engineering field, it brings various desires and hopes for the scientific community to reproduce and modify the defects within the gene of interest for the enhancement, even in large animals including livestock. Heo et al. present the CRISPR/Cas9 nuclease-based altering in animals, particularly in bovine. They have generated iPSCs (induced pluripotent stem cells) by exogenously introducing Yamanaka factors and GSK3β and MEK inhibitor in bovine somatic fibroblasts through CRISPR/Cas9 nuclease-based homologous recombination to generate naïve pluripotent stem cells [126]. Editing through CRISPR/Cas9 nuclease further paves the path to the door of future precise and efficient editing tools for gene editing in large animals.

## 9. Disease-Resistant Animals

Lately, the CRISPR system has developed as a reliable method for bringing about disease resistance to animals within the agricultural field. Recently, CRISPR/Cas9 nickase mediated exogenous knock-in of NRAMP1 in BFFs (bovine fetal fibroblast) was carried out to generate tuberculosis-resistant genetically modified cattle. They have created nine cows that exhibited resistance to tuberculosis [109]. Furthermore, another gene, PRNP, encodes PrPSc, an infectious protein that causes fatal disorders in cattle and humans such as bovine spongiform encephalopathy, Creutzfeldt–Jakob disease, and chronic wasting disease (in cervids). CRISPR/Cas9 has been utilised to generate knockouts in both bovine foetal fibroblasts and early embryos by precisely editing bovine PRNP coding exon 3 specifically [127].

Another IARS (Isoleucyl-tRNA synthetase) syndrome is a recessive disorder in Japanese black cattle affected by a single nucleotide substitution mutation. Rectifying the mutation CRISPR innovation was applied to incorporate a donor DNA-containing synonymous codon (Aequorea coerulescens Green Fluorescent Protein) to correct the amino acid arrangement or sequence [128].

Johne’s diseases (JD) and chronic enteritis in cattle are caused by the microbe Mycobacterium avium subspecies paratuberculosis (MAP). SNPs in the interleukin-10 receptor alpha gene (IL10RA) relate to MAP infection and mastitis in dairy cattle. This gene codes for the alpha chain of the IL-10 receptor, whose ligand, IL-10, functions as a critical regulator of inflammation and has been linked to MAP disease pathogenesis. In addition, the CRISPR/Cas9 gene editing tool was used to create an IL10RA knock-out in MAC-T cells, which was established from bovine mammary epithelial cells. The findings demonstrate the extensive and critical effects of a knock-out of the IL10RA gene in modifying pro-inflammatory cytokine expression and assisting the immunoregulatory component of IL10RA in inducing an anti-inflammatory response, as well as its possible functional interaction affiliation among immune responses linked to JD [129].

Brucellosis is one of the foremost grave zoonotic diseases globally. Its seriousness is not constrained to the animal’s populace, as it also causes a critical finance-related burden for stockbreeders. Recently, Karponi et al. transduced infected cells with lentiviral vectors containing the CRISPR/Cas9 gene editing system to inactivate a gene involved in Brucella replication within host cells, specifically the virulence-associated gene virB10 or RpolA (RNA polymerase subunit A). They reported that on the first and fourth days after transduction with the CRISPR/Cas9 vector against bacterial RpolA at a multiplicity rate infection (MOI) of 60, the number of internalised brucellae/cells is drastically reduced [130].

## 10. Improving Animal Welfare

In modern dairy cattle farming, a plethora of bovines have a horned phenotype. It increases the risk of injury or damage to the animal as well as to agriculturists. A polled phenotype is preferred in this case. Usually, polled phenotypes are essentially used in Angus meat breeds. In different meat breeds, the polled Celtic (Pc) variation, a 202 bp indel wide array within the polled locus, induces a polled characteristic. Previously, transfecting TALEN in the form of mRNA into bovine fibroblasts combined with an HDR prototype bearing the 202 bp indel mutation resulted in a genetic sibling with a polled morphology. They used the CRISPR/Cas12a framework as a novel and efficient approach for incorporating the Pc variety into the genome of a high-performance HF breeding bull in their project. The polled Celtic variation was successfully introduced into the genome of a horned HF using the CRISPR/Cas12a technique, allowing the breeding bull to be created with a polled phenotype and thereby eliminating the need for dehorning [131].

## 11. Improving Productive Traits

The peroxisome proliferator-activated receptor (PPAR), a positive regulator of adipogenesis, is expressed within adipose tissue. Without PPAR expression, adipocytes are unable to initiate the adipogenic differentiation process. PPAR expression in adipocytes comes before other lipid-related gene expressions during adipogenic differentiation. MSTN, on the other hand, is a muscle development negative regulator that inhibits muscle cell multiplication and differentiation, as well as muscle development and formation. As a result, this research on using CRISPR/Cas9 to achieve myostatin point mutation and PPAR site guided knock-in within the bovine genome is remarkable [132].

Semen sexing and desire descendant birth of livestock may be a critical point to attain recently. Non-radioactive hybridisation, histocompatibility-Y antigen, and in-situ fluorescence hybridisation, sex chromosome-based PCR investigation, and labelled Y-specific in situ probes have also been used to image the sex of developing life or embryos before foetus transition in animals. By knock-in eGFP (green fluorescent protein (constitutively expressed fluorescent)) gene within the Y-chromosome of bovine fetal fibroblast (BFF) cell lines with the assistance of CRISPR/Cas9 and afterwards Y-Chr-eGFP transgenic BFF cell’s nucleus transferred by SCNT strategy. Y-Chr-eGFP transgenic BFFs nucleus containing foetus are effectively visualised the green fluorescent though eGFP reporter shown Y-containing embryo. Semen is sexing in this straightforward manner by just monitoring colour tracer (eGFP) as all XY embryos present the green colour beneath the fluorescence microscopy, whereas XX having an embryo does not show the green colour [133].

Livestock is gradually diminishing with time since different variables; the low fertility rate could be a significant concern. It is mandated to understand the female animal reproductive physiology, reproduction cycle, and associated molecular network to overcome these issues. Exogenous TSP1 (thrombospondin 1) gene molecular candidate dependable for governing the rhythm of the reproductive cycle of the buffalo. Its expression impacts the steroidogenic function of luteal cells in vitro. Interestingly, exogenous TSP1 is potentiated to increase caspase 3, whereas diminishes practical luteal cells. To improve the viability of luteal cells, decrease caspase 3 activations, and increment progesterone generation, knock-out of TSP1 was essential to illustrate the activities mentioned. Utilising CRISPR innovation benefits, one research group genetically modified the TSP1 gene in bubaline luteal cells. Afterwards, they have demonstrated that endogenous TSP1 acts as a negative controller for angiogenesis, which reduces progesterone production and number the luteal cells through apoptosis at the time of luteal regression [134]. This finding can benefit the future scientific community by encouraging them to consider and improve or stretch the female animal’s regenerative cycle.

## 12. Legal and Ethical Issues for the Adoption of CRISPR Technology

Along with the positive aspects of genome editing technology, there are a number of drawbacks that should not be overlooked. We have gone through some of the most important bioethical and legal concerns around genetically engineered farm animals. As we all know, genome editing methods have resulted in profound changes in molecular biology science. CRISPR/Cas9 has emerged as the most prevalent gene editing technique due to advantages such as ease of use, high precision, and low cost compared to older technologies such as ZFN and TALEN. Because of these advantages, CRISPR/Cas9 technology is easily applicable in any molecular biology laboratory. The rapid emergence of CRISPR-Cas9, on the other hand, has created new legal, bioethical, and social challenges in agriculture, medicine, livestock, and the environment.

On the other hand, CRISPR/Cas9 technology’s bioethical consequences for the environment, agriculture, and animals should not be disregarded [135]. Here, we sought to summarise the risks and bioethical concerns related to CRISPR/Cas9. Animal welfare arguments are used to support and oppose genome editing in cattle. For example, genome editing has recently been utilised to create hornless calves, obviating the need for the painful dehorning routinely performed in the agricultural industry to protect both cows and farmers from injury [131]. On the contrary, it is also claimed that this goal may be reached in other ways: instead of developing polled animals, the growing environment of cattle may be modified to reduce accidents, horn coverings might be used, or dehorning may be done under anaesthetic.

Additionally, genome editing has the potential to improve animal health and welfare by making animals more disease resistant or adaptable to changing environments [136]. On the other side, such genome editing applications were thought to allow for even more farming intensification, such as developing polled or disease-resistant animals that could be housed at higher densities. While these authors claimed that increased agricultural intensity would lessen animal suffering, others questioned if this was possible given existing trends of firms improving animal welfare [90,131]. Furthermore, some experts say that modifying the genomes of agricultural animals to enhance production efficiency might cause secondary consequences that are harmful to animal welfare. Increased muscle growth, for example, might lead to more Caesarean sections, limb issues, or respiratory difficulties [136,137,138]. Finally, genome editing might have a variety of additional effects on animal welfare. The scientists warned that employing somatic cell nuclear transfer (SCNT) cloning to deliver the nuclease-mediated modifications might negatively affect animal welfare; SCNT has been associated with embryonic loss, postnatal mortality, and congenital disabilities [136]. According to scientists, genome editing might result in off-target mutations or unintended repercussions, both of which might be hazardous to animal health. Because gene drift persists in a population, off-target mutations will continue to emerge in each generation [139,140,141].

Furthermore, as generations pass, the number of mutations and their effects may increase [133]. Others suggested that genome editing using tailored nucleases would result in more minor off-target effects than previous approaches. The use of CRISPR/Cas9 to generate desired genetic modifications makes it exceedingly difficult to detect and regulate genetically modified organisms (GMOs) on the market once they leave the lab. As a result, regulatory bodies such as the Food and Drug Administration (FDA) in the United States and the European Medicines Agency (EMA) should investigate whether GMOs are safe for consumers [135]. Nevertheless, it is unclear how to assess the potential of a burgeoning business with CRISPR/Cas9.

Genome editing is distinct from older genetic engineering techniques, relying on the intracellular use of artificial nucleases created by a researcher. Some off-target mutations may be deleterious and have a negative impact on animal health, raising concerns about animal welfare. As a result, a greater focus on animal welfare by lowering the risk of off-target mutations may increase public trust and, eventually, social acceptance of products derived from genome-edited livestock. Off-target mutations can be detected using three methods: sequencing only potential off-target sites, whole-genome sequencing (WGS), and whole-exome sequencing (WXS). As a report on bovine genome editing demonstrated, it is currently appropriate to investigate off-target mutations in animal embryos or somatic cells as thoroughly as possible [137].

**Figure 6:**
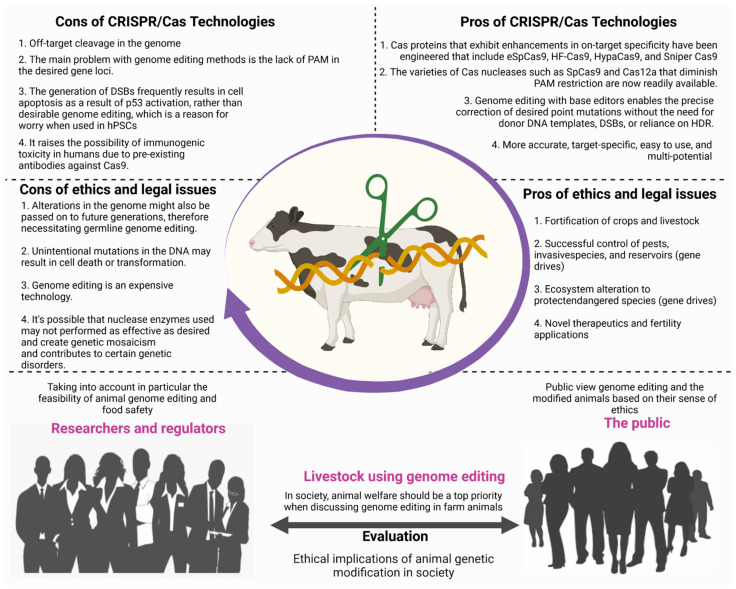
This illustration depicts the interaction between the public, scientists (or researchers), and regulatory agencies (regulators). Additionally, this figure illustrates the pros and cons of technology in terms of technical, ethical, and legal issues.

Rapid advances in livestock genome editing research suggest that animal products will be available for purchase soon after a country’s food safety has been established. Previous debates over genetically modified animals and animal cloning, on the other hand, highlight the importance of people’s ethical sensibilities as well as animal welfare (Figure 6). The use of genome editing in farm animals should be done only after careful consideration of animal genetic modification’s social and ethical implications. Furthermore, for animal welfare reasons, developers should thoroughly investigate the occurrence of off-target mutations in the breeding of genome-edited animals. If regulators want to increase public acceptance and avoid major societal disagreements, they should question developers about off-target mutations and encourage public discussion about livestock breeding using genome editing. Such farm animal products will never be accepted unless genome editing animals’ practical and ethical implications are taken into account.

The worldwide CRISPR gene editing industry is projected to reach $10.82 billion by 2030, according to estimates. Research market on the CRISPR gene editing technology include different product type—CRISPR Products: Kits and Enzymes (both vector-based and DNA-free Cas9), Library resources, Software Applications, CRISPR/Cas9 other services. Countries participating are North America—namely the United States and Canada—is the most often targeted region. Europe—Germany, France, Italy, the United Kingdom, Spain, and Switzerland. Australia, China, Japan, India, Singapore and South Korea are among the countries in the Asia-Pacific region, https://www.globenewswire.com/news-release/2021/02/01/2167183/0/en/Global-CRISPR-Gene-Editing-Market-Focus-on-Products-Applications-End-Users-Country-Data-16-Countries-and-Competitive-Landscape-Analysis-and-Forecast-2020-2030.html. (accessed on 10 June 2021)

Considering those abovementioned ethical and legal aspects from the public, numerous benefits outweigh the drawbacks, implying that any country should allow CRISPR practices. CRISPR genome editing should be used to improve benign application, such as improve productivity (milk and meat), animal health (infectious disease outbreak) and enhance the welfare of animals (dehorning of cattle) due to its benefits, including the precision, specificity, efficiency and cost-effectiveness of the technology. Of course, learning and perfecting any new technology takes time. It will be critical to ensure that a guide RNA is specific for its target gene so that the CRISPR system does not attack other genes by accident. Before CRISPR therapies can be widely used in medicine, it will be necessary to find a way to deliver them into the cell or embryo. Even though there is still much to learn, CRISPR has proven to be an invaluable research tool. Indeed, there is enough interest in the field to warrant the formation of several biotech start-ups that aim to treat human and animal diseases using CRISPR-inspired technology. CRISPR/Cas9 has been used in many nations to delete genes in livestock to increase muscle mass, eradicate disease, improve animal welfare, and increase hair growth to increase the stock size for the country’s commercial meat and wool industries. This could become a common way to expand livestock industries in the future.

## 13. Conclusions

With its relative ease of implementation, the CRISPR/Cas9 device outperforms other mutagenic approaches and can alter DNA with higher efficiency than current technologies like ZFN and TALEN. Consequently, its rapid progression has fuelled plenty of other new concepts for solving current livestock challenges. It is mainly for improving animal health, characteristics, welfare, and their association with environmental preservation and impacts on human health. The CRISPR/Cas9 system’s extensibility enables the rapid development and testing of different pharmacological strategies, resulting in shorter production times over other genetic engineering methods. This innovation permits shaping the animal kingdom and the environment as never accomplished before to seek human purposes to upgrade worldwide well-being.

## Figures and Tables

**Table 1:** Computational tools are available for the design of sgRNAs to evaluation of guide RNA efficiency and prediction of its specificity. All the links were accessed on 7 June 2021.

Evaluation Guide RNA Efficiency	Link to Access the Algorithms
E-CRISP (Cas9)	http://www.e-crisp.org/E-CRISP/
CRISPRscan (Cas9, Cpf1)	https://www.crisprscan.org/gene/
evaluateCrispr (Cas9)	https://eu.idtdna.com/site/order/designtool/index/CRISPR_SEQUENCE
sgRNAScorer (Cas9, Cpf1)	https://sgrnascorer.cancer.gov/
SSC (Cas9)	http://cistrome.org/SSC/
WU-CRISPR (Cas9)	http://crisprdb.org/wu-crispr/
Azimuth (Cas9)	https://github.com/MicrosoftResearch/Azimuth
CRISPRater (Cas9)	http://www.leukemia-research.de/resources/crisprater/
CRISPRpred (Cas9)	https://bmcbioinformatics.biomedcentral.com/articles/10.1186/s12859-020-3531-9
CASPER (Cas9, Cpf1)	https://pubmed.ncbi.nlm.nih.gov/28968798/
DeepCpf1 (Cpf1)	http://deepcrispr.info/
TSAM (Cas9)	https://pubmed.ncbi.nlm.nih.gov/29672669/
TUSCAN (Cas9)	https://github.com/BauerLab/TUSCAN
uCRISPR (Cas9)	https://github.com/Vfold-RNA/uCRISPR
**Predict guide RNA specificity**	
CasOT (Cas9)	http://eendb.zfgenetics.org/casot/
Cas-OFFinder (custom)	http://www.rgenome.net/cas-offinder
sgRNAcas9 (Cas9)	http://www.biootools.com/
FlashFry (custom)	https://aaronmck.github.io/FlashFry/
Crisflash (Cas9)	https://github.com/crisflash/crisflash
MIT (Cas9)	https://crispr.mit.edu
CCTop (Cas9, Cpf1)	https://cctop.cos.uni-heidelberg.de:8043/
CFD (Cas9)	https://www.genscript.com/gRNA-detail/mouse/11537/Cas9/Cfd-CRISPR-guide-RNA.html
CRISPRoff (Cas9)	https://www.genscript.com/gRNA-detail/mouse/11537/Cas9/Cfd-CRISPR-guide-RNA.html
uCRISPR (Cas9)	https://github.com/Vfold-RNA/uCRISPR
CRISTA (Cas9)	https://crista.tau.ac.il/
Elevation (Cas9)	https://github.com/microsoft/Elevation
DeepCRISPR (Cas9)	http://deepcrispr.info/DeepSpCas9/

## Data Availability

The study did not report any data.
